# Supplementary figures and images for: Benidipine impairs innate immunity converting sublethal to lethal infections in a murine model of spotted fever rickettsiosis
Source: PLoS Negl Trop Dis. 2024 Feb 26;18(2):e0011993. doi: 10.1371/journal.pntd.0011993 (PMC10919851; doi:10.1371/journal.pntd.0011993)

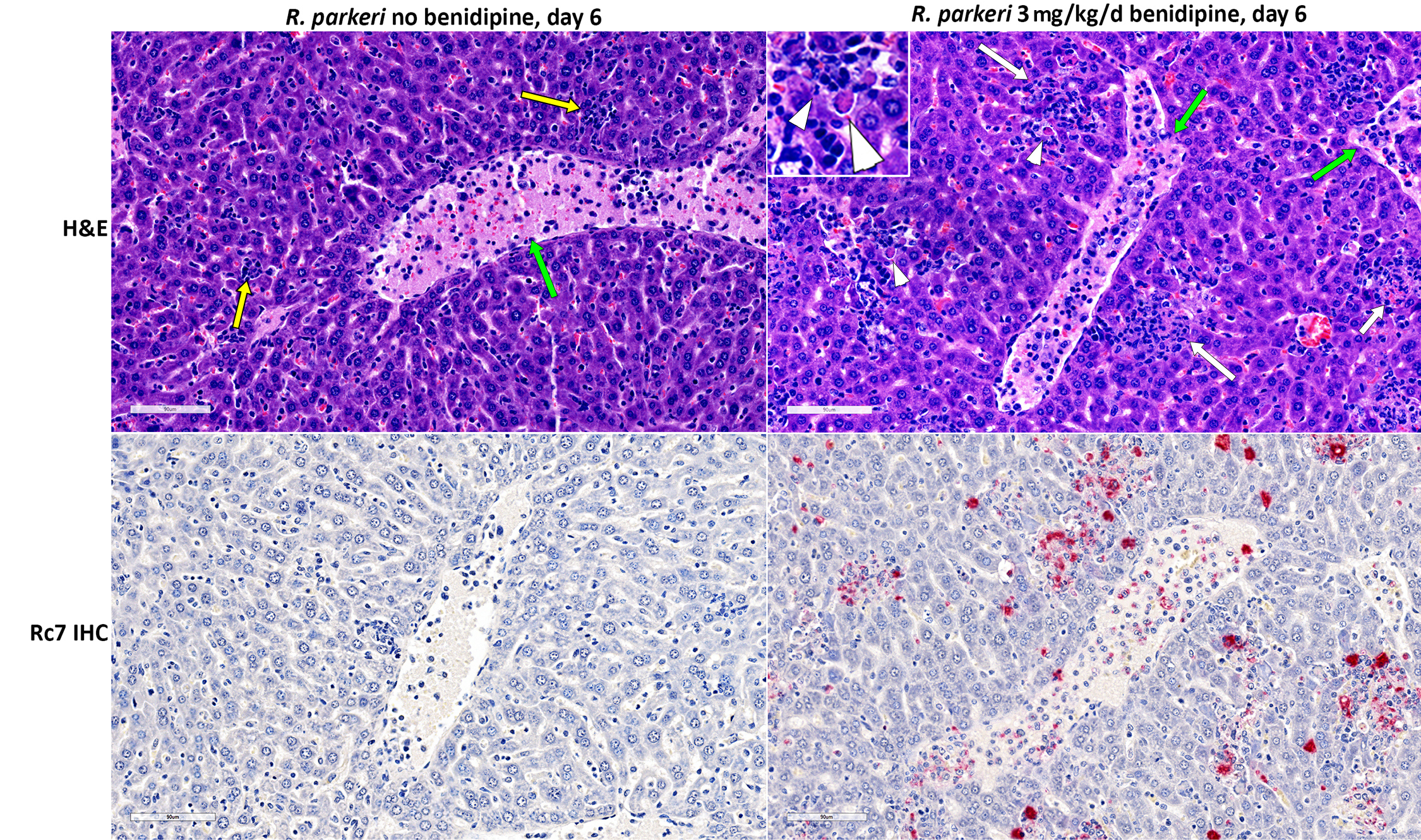

Supplement: S1 Fig — High resolution image comparison of hepatic pathology on day 6 after R. parkeri infection with no benidipine (left panels) and with 3 mg/kg/d benidipine (right panels). The top left H&E stain panel demonstrates the waning lobular and periportal inflammatory infiltrates with focal portal vein thrombosis (green arrow) and small granuloma-like aggregates (yellow arrows) in untreated infected animals, whereas the infected treated animals were more likely to have persistent lobular aggregates (white arrows) of inflammatory cells that included both mononuclear cells and neutrophils, as well as necrotic and apoptotic cells (white arrowheads and insert) and more frequent portal vein thromboses (right panel, green arrows). The bottom panels demonstrate the rare presence of R. parkeri by IHC (Rc7 IHC) in the livers of infected untreated animals (bottom left), but an inability to control the infection in animals treated with benidipine. Bar = 90 μm. (TIF) [file pntd.0011993.s001.tif]

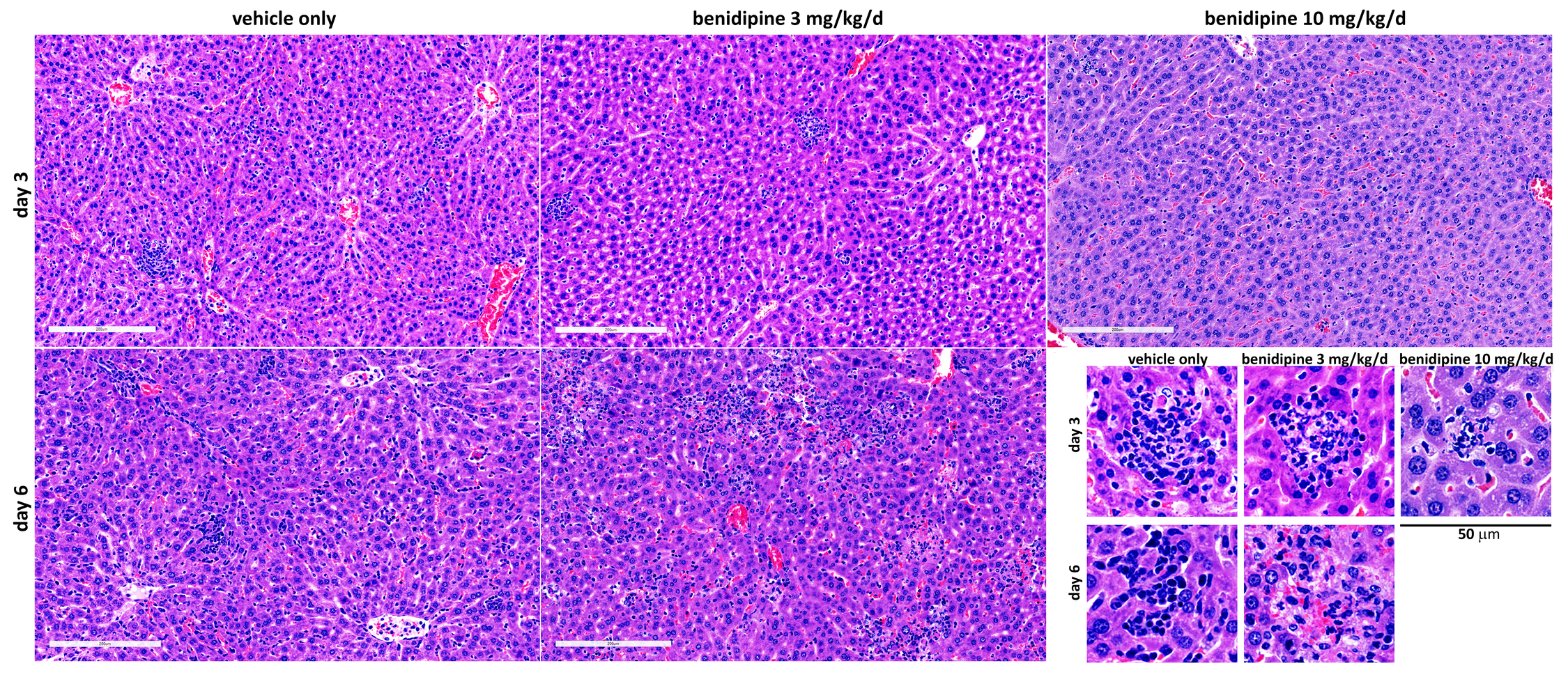

Supplement: S2 Fig — High resolution H&E-stained images of liver from R. parkeri-infected mice treated with vehicle only, or with 3 or 10 mg/kg/d benidipine. Note the lobular hepatitis with nodular mononuclear cell inflammatory infiltrates of similar distribution and intensity in untreated and mice treated with 3 mg/kg/d benidipine on day 3 and worse on day 6. Note also the diminished lobular hepatitis on day 3 in animals treated with 10 mg/kg/d benidipine. The inserts demonstrate the differences in inflammatory cell content in the nodular infiltrates in the hepatic lobules where treatment with benidipine results in the supplemental recruitment of neutrophils with greater tissue necrosis compared to untreated infected mice. (TIF) [file pntd.0011993.s002.tif]

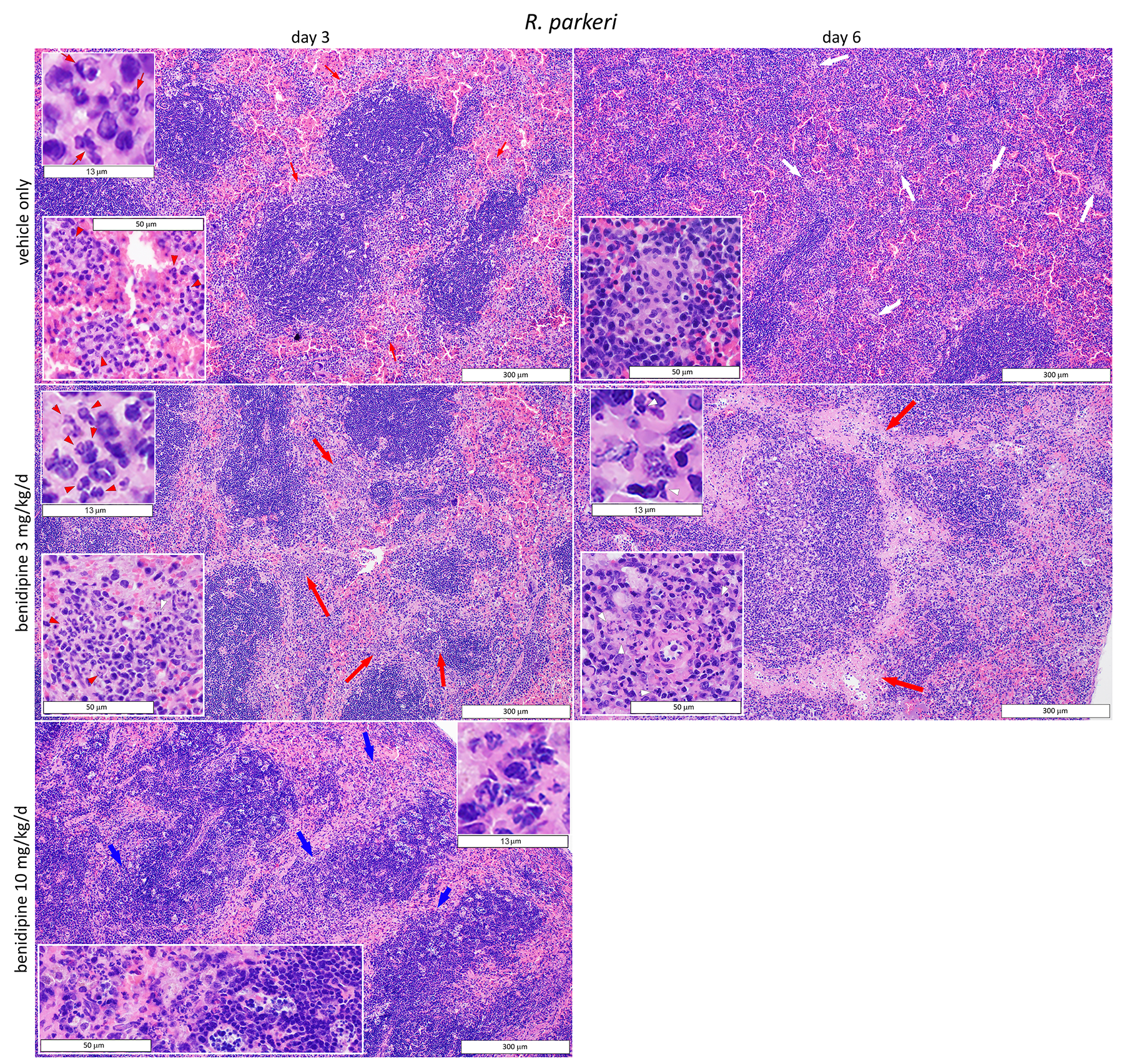

Supplement: S3 Fig — High resolution H&E-stained images of spleen from R. parkeri-infected mice treated with vehicle only, or with 3 or 10 mg/kg/d benidipine. Note the retained by fragmenting follicles and white pulp with increasing cellularity in the red pulp of infected untreated animals at day 3, followed by the extensive loss of white matter and follicular architecture and massive expansion of red pulp by mononuclear cells in untreated animals at day 6. The red arrows and insert demonstrate small neutrophilic and necrotic regions within the red pulp at day 3, and the resolution of these foci by reparative granuloma-like foci (white arrows and insert) at day 6 when R. parkeri burden is at a nadir by PCR and IHC. In contrast, infected animals treated with benidipine had significantly less red pulp expansion and worse fragmentation of white pulp and follicles with clear evidence of substantial neutrophilic infiltration and tissue/cellular necrosis (red arrows 3 mg/kg/d benidipine, blue arrows 10 mg/kg/d benidipine, and inserts). Also note the extensive phagocytosis of cellular and nuclear debris in macrophages within follicles with benidipine (insert day 3, benidipine 10 mg/kg/d). By day 6 with animals receiving 3 mg/kg/d benidipine, there was extensive red pulp necrosis (red arrows and insert) lacking any evidence of tissue repair at a time when R. parkeri burden in the spleen is at peak. (TIF) [file pntd.0011993.s003.tif]

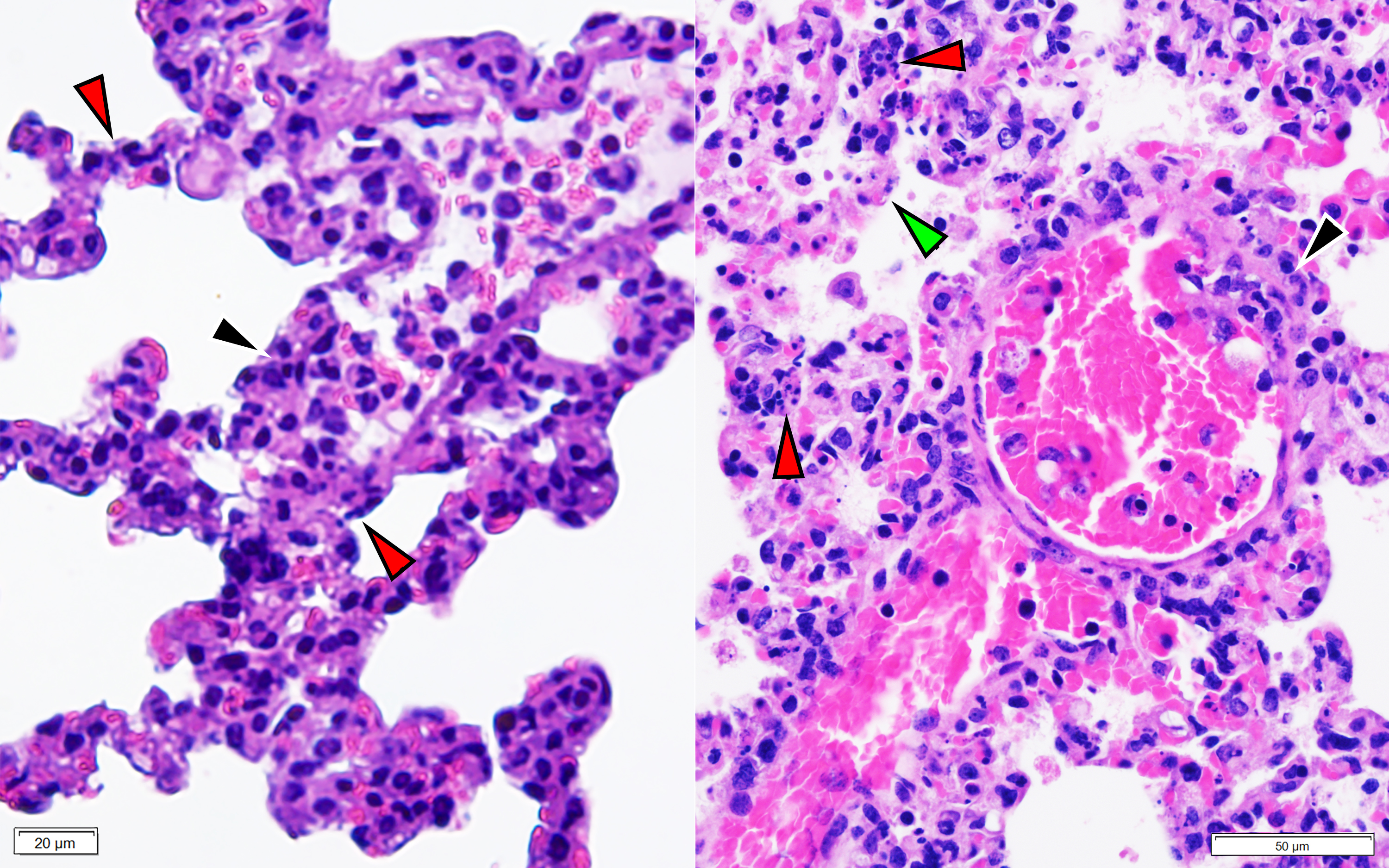

Supplement: S4 Fig — Representative high resolution H&E stained image of lung from and R. parkeri-infected mouse treated with vehicle only (left panel) or treated (right panel) with 3 mg/kg/d benidipine and necropsied on day 6 p.i. Note the dense mixed inflammatory cell interstitial infiltrates that widen alveolar septa, the segmental vasculitis of pulmonary venules (black arrows), capillaritis with karyorrhectic debris (red arrows), and focal alveolar wall necrosis in the treated animal (green arrow). (TIF) [file pntd.0011993.s004.tif]

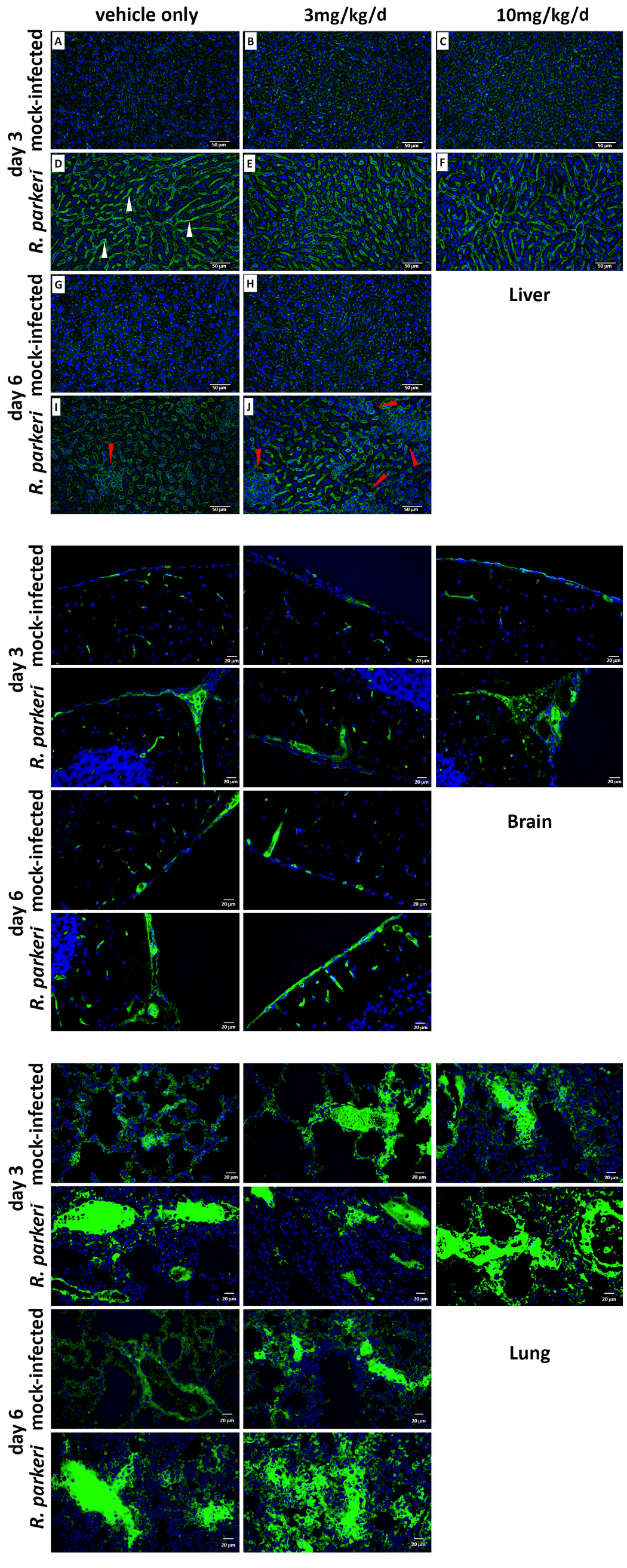

Supplement: S5 Fig — The vascular leakage images that were at the median-rank for each tissue, time p.i., infection or mock infection, and drug dose are shown. For dextran extravasation measurements from liver and brain, the percent area of fluorescence was measured over the entire image. For the lungs, large vascular structures were excluded from the fluorescent images to offset intravascular retention fluorescent signal. Note the minor variations over time, infection status and drug treatment, none of which were reproducibly significant. (TIF) [file pntd.0011993.s005.tif]

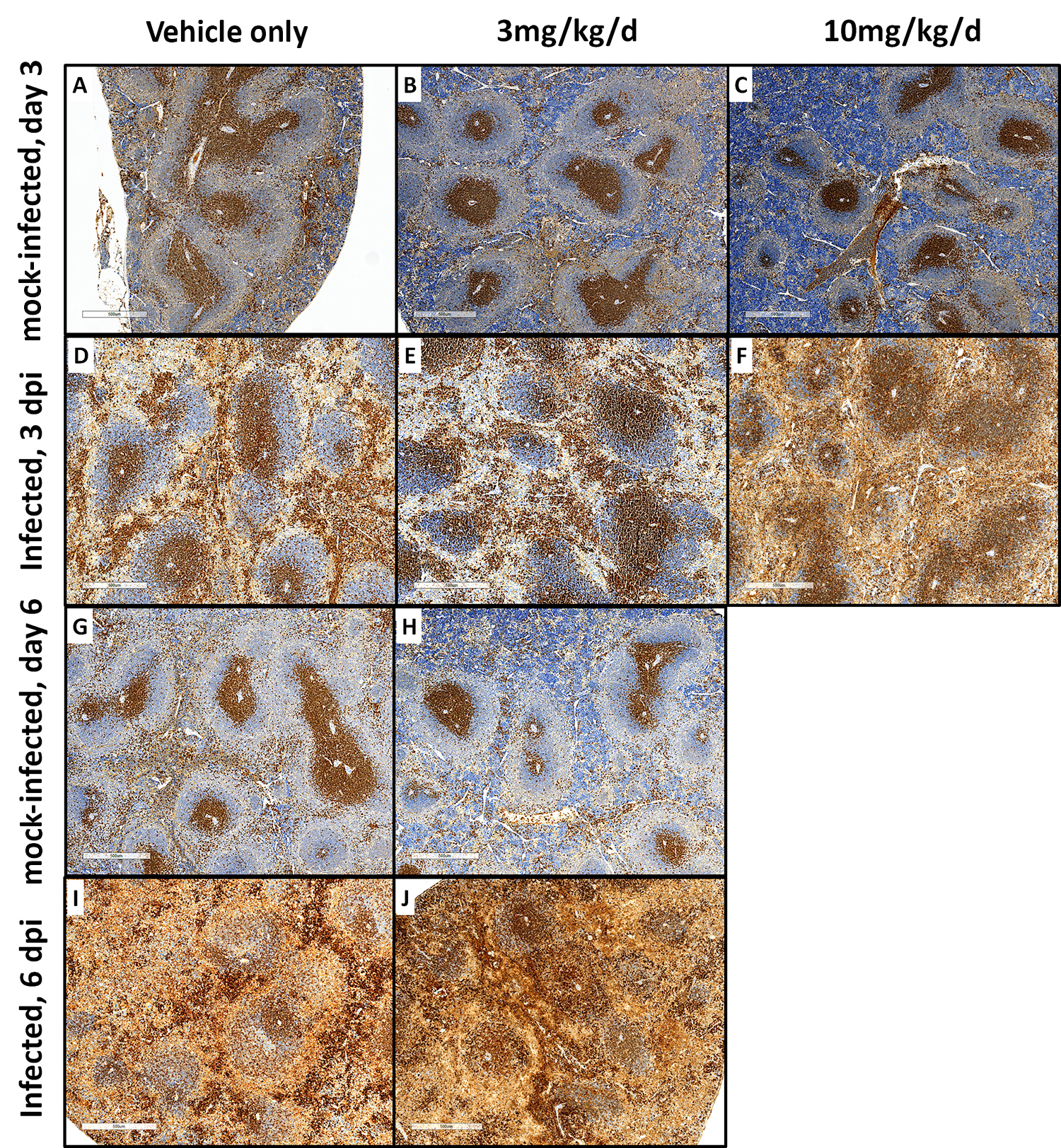

Supplement: S6 Fig — Representative CD3 IHC on splenic tissue from R. parkeri-infected and mock infected animals at days 3 and 6 d.p.i. with or without benidipine treatment as indicated. Note the highest density in uninfected animals within the white pulp and TCZ/PALS, and the marked increase in CD3 cells in the red pulp of infected animals. Bar = 500 μm. (TIF) [file pntd.0011993.s006.tif]

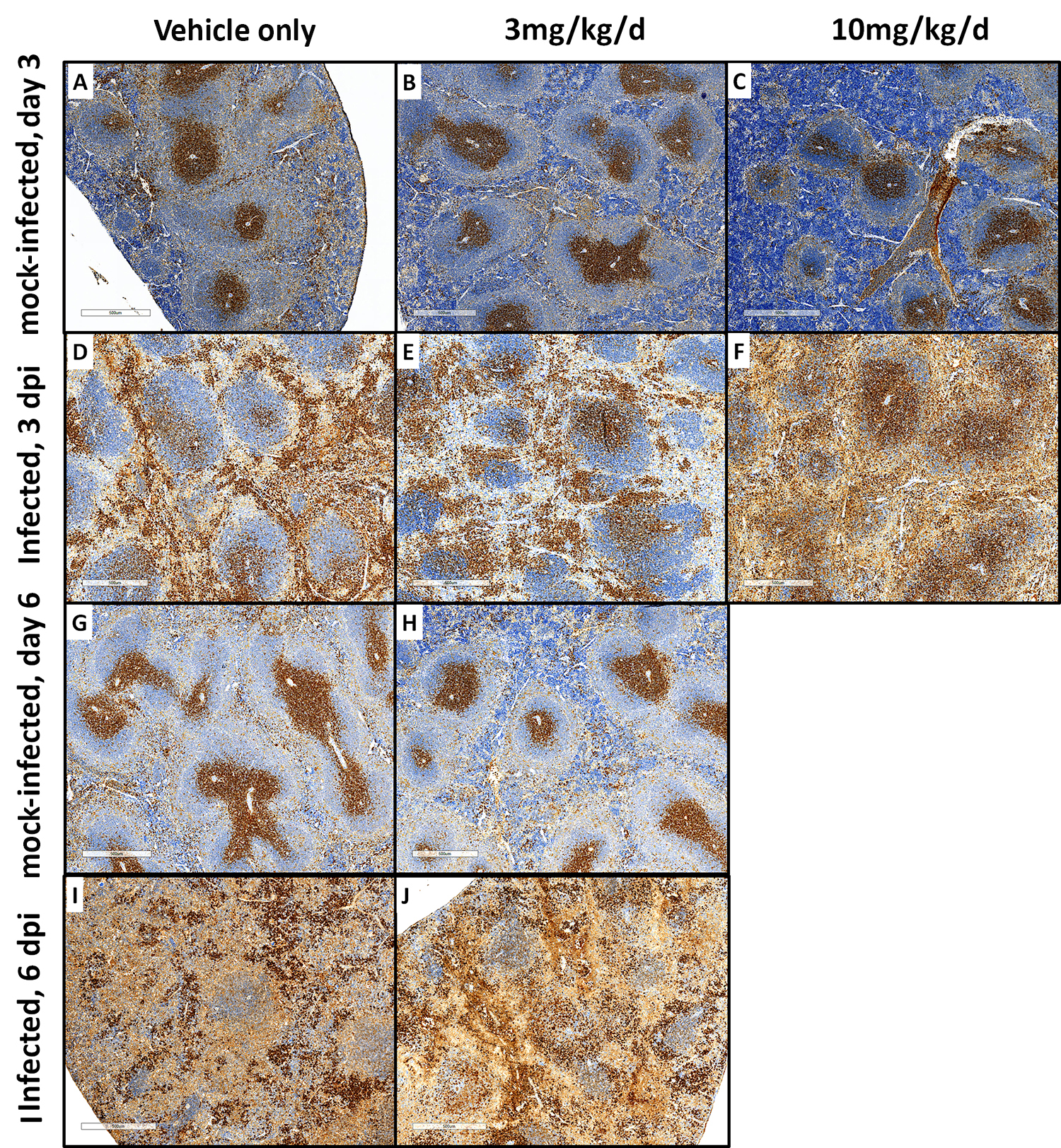

Supplement: S7 Fig — As for CD3 cells, note the predominant restriction of CD4 cells to the white pulp follicles and TCZ/PALS in uninfected animals, and the loss from the regions accompanied by a simultaneous increase in CD4 cells in the red pulp of infected animals. Note that benidipine treatment in infected mice resulted in less white pulp depletion of CD4 cells and a diffuse distribution of CD4 cells throughout splenic parenchyma compared to the clustered appearance in the red pulp with infection but no benidipine. Bar = 500 μm. (TIF) [file pntd.0011993.s007.tif]

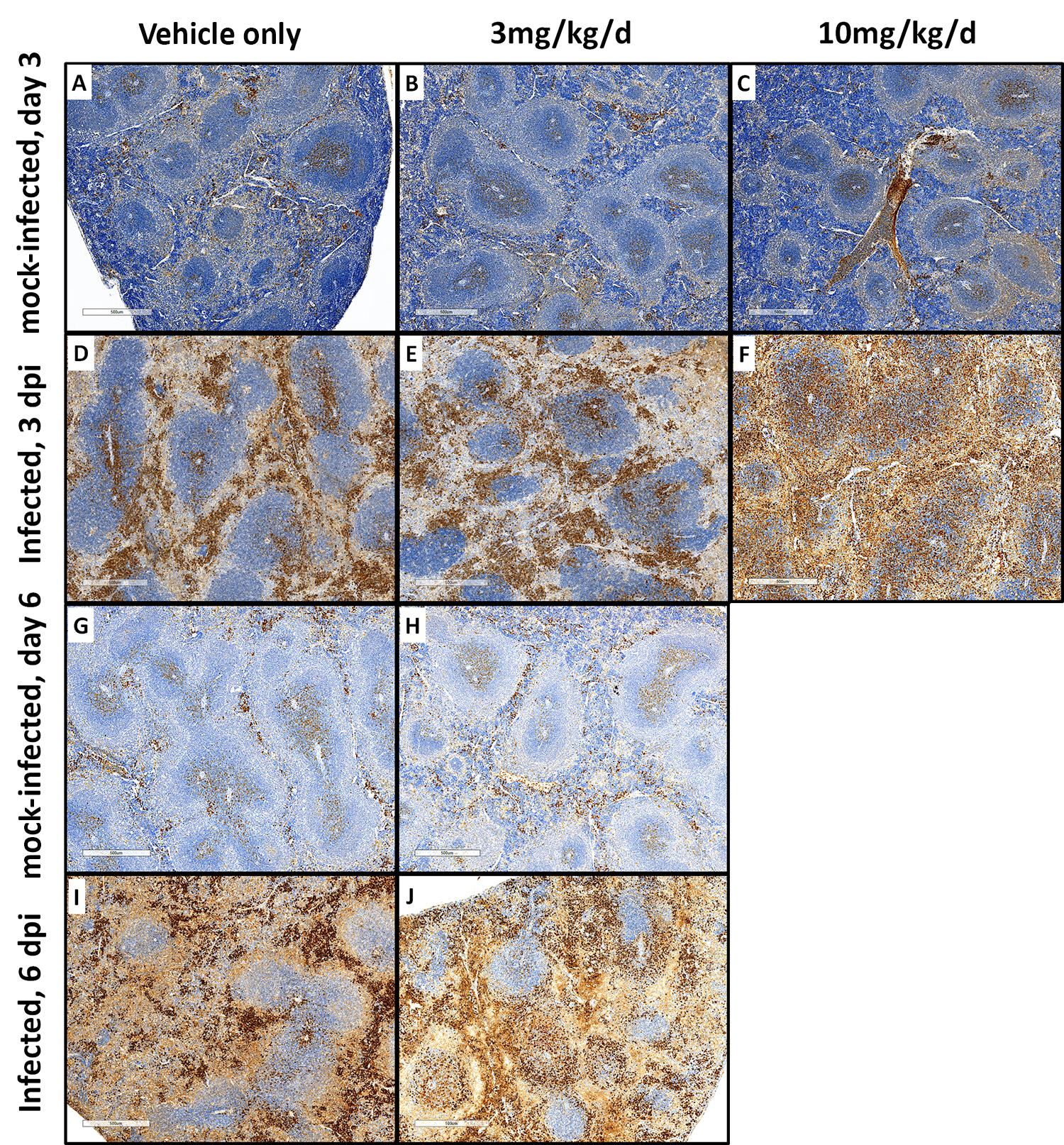

Supplement: S8 Fig — Note the scant distribution of CD8 cells in the red pulp of uninfected animals compared to the marked increase in red pulp CD8 cells with infection, as well as the diffuse distribution, including within white pulp follicles, with benidipine treatment and infection. Bar = 500 μm. (TIF) [file pntd.0011993.s008.tif]

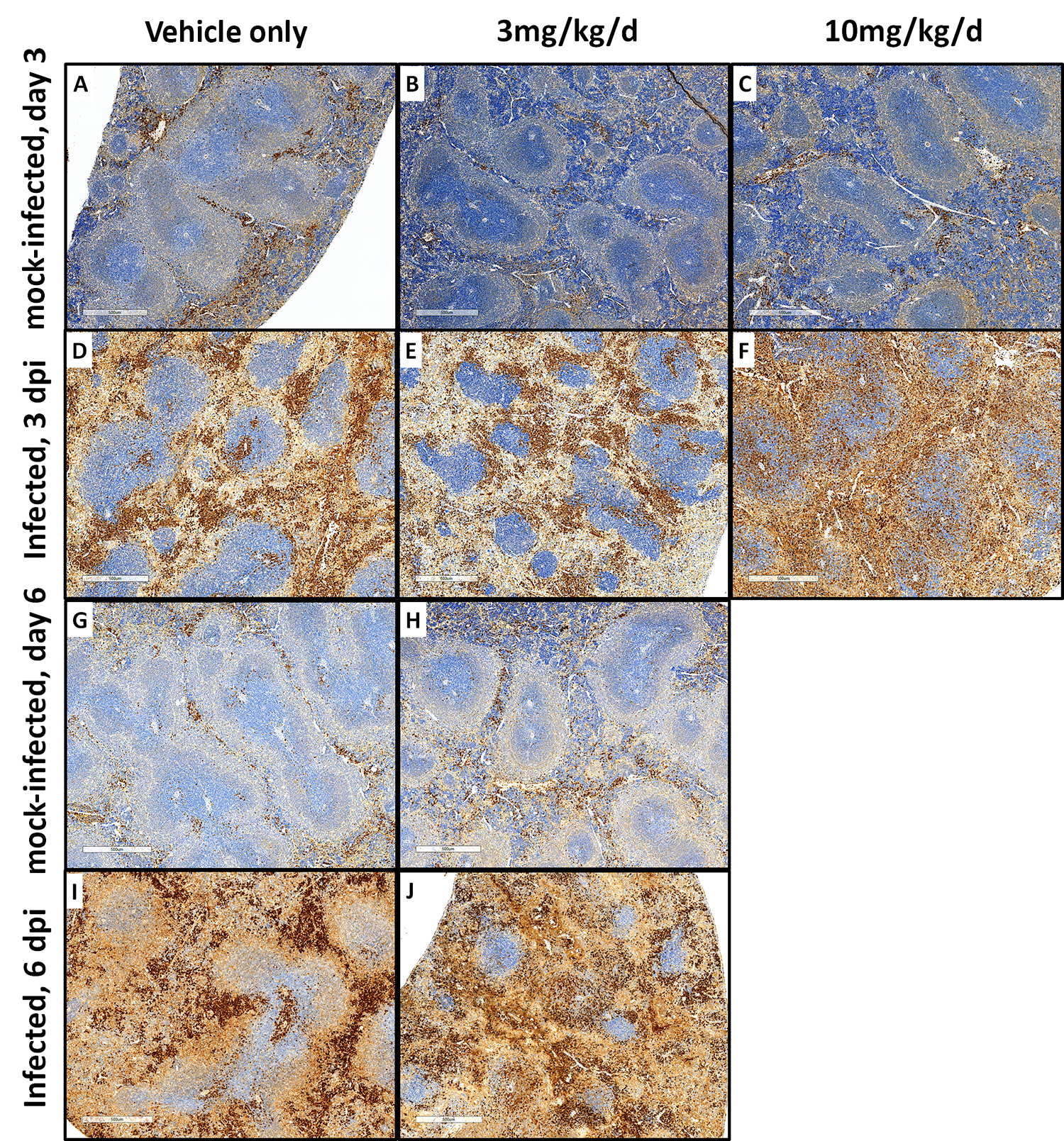

Supplement: S9 Fig — Note the discrete localization of NCR1+ cells within the red pulp of uninfected mice, and the marked increase in numbers with infection. With benidipine treatment in infected mice, the localization of NCR1+ cells became more diffuse, including incursion into white pulp follicles and TCZ/PALS. Bar = 500 μm. (TIF) [file pntd.0011993.s009.tif]

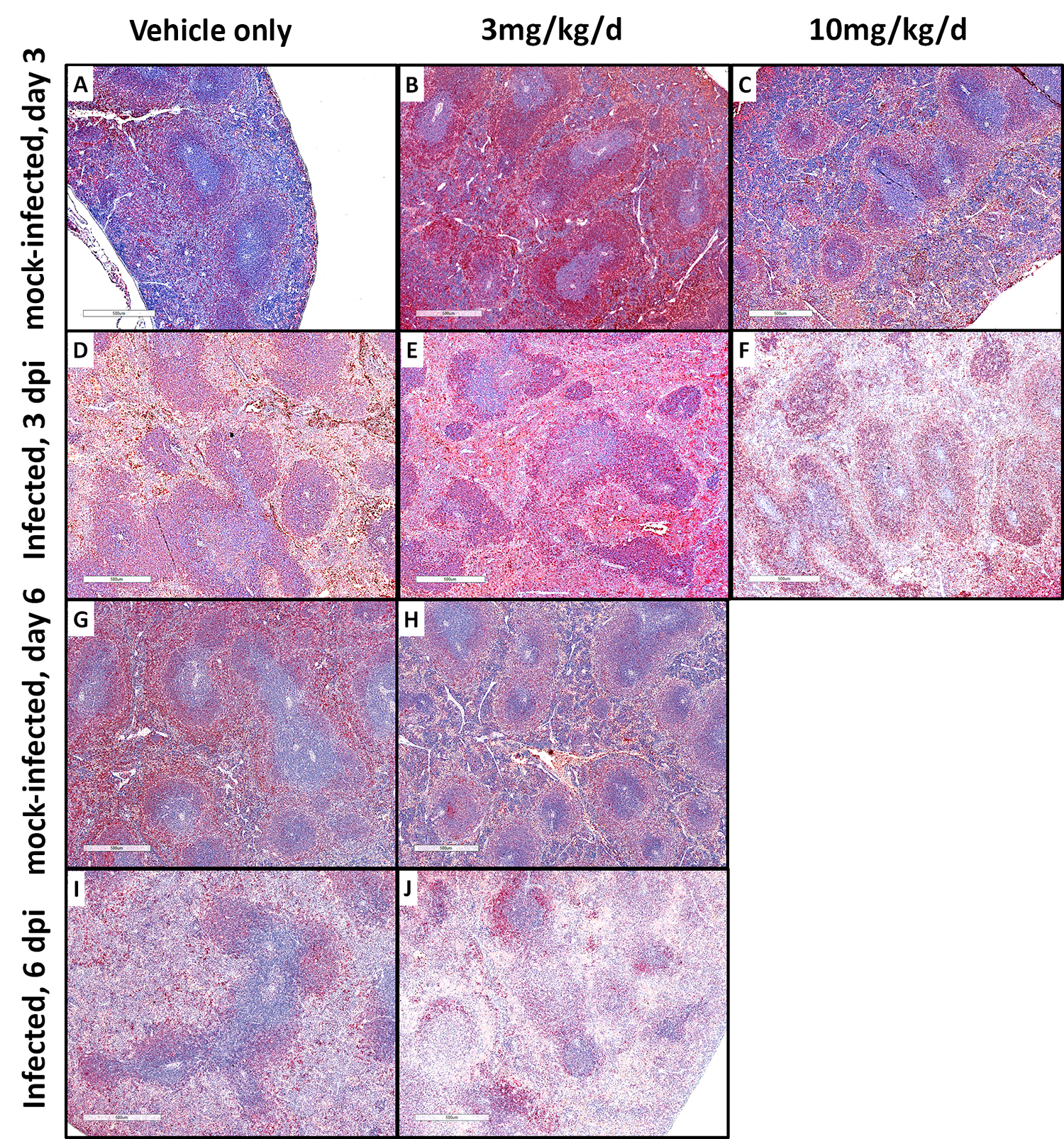

Supplement: S10 Fig — Note the localization of B220+ cells primarily in marginal zones around the white pulp follicles in uninfected animals, and the more diffuse distribution into the red pulp with benidipine. Total quantity of B220+ cells does not dramatically change through these conditions. Bar = 500 μm. (TIF) [file pntd.0011993.s010.tif]
