# Supplementary material for: Benidipine impairs innate immunity converting sublethal to lethal infections in a murine model of spotted fever rickettsiosis
Source: PLoS Negl Trop Dis. 2024 Feb 26;18(2):e0011993. doi: 10.1371/journal.pntd.0011993 (PMC10919851; doi:10.1371/journal.pntd.0011993)
Supplement: Attachment — Submitted filename: 240205 PNTD-D-23-01130 R2 Response to Rev.pdf [file pntd.0011993.s013.pdf]

UNIFORMED SERVICES UNIVERSITY OF THE HEALTH SCIENCES
F. EDWARD HEBERT SCHOOL OF MEDICINE
America's Medical School
4301 JONES BRIDGE ROAD
BETHESDA, MARYLAND 20814-4799
www.usuhs.edu

February 5, 2024

Bin Gong, Ph.D., M.D.
Guest Editor
PLOS Neglected Tropical Diseases

Joseph Vinetz
Section Editor
PLOS Neglected Tropical Diseases

Re: PNTC-D-23-01130, 2nd revision submission

Dear Drs. Gong and Vinetz,

On behalf of my coauthors, I'm pleased to submit the revised version of our manuscript entitled "Benidipine impairs innate immunity converting sublethal to lethal infections in a murine model of spotted fever rickettsiosis." This letter details the list of responses (in italics) to all reviewer comments, including all changes made to the manuscript since the R1 version. Also uploaded with this letter are the full revised version, the revised version with track changes, as well as the revised figures and images, in accordance with reviewer suggestions and recommendations.

Thank you for your attention to the manuscript. We look forward to subsequent feedback.

Respectfully,

J. Stephen Dumler, M.D.
Professor and Chairperson
Uniformed Services University of the Health Sciences
School of Medicine
Department of Pathology
john.dumler@usuhs.edu

Reviewer's Responses to Questions

Key Review Criteria Required for Acceptance?</br></br>
As you describe the new analyses required for acceptance, please consider the following:</br></br>

Methods</br></br>
-Are the objectives of the study clearly articulated with a clear testable hypothesis stated?</br>
-Is the study design appropriate to address the stated objectives?</br>
-Is the population clearly described and appropriate for the hypothesis being tested?</br>
-Is the sample size sufficient to ensure adequate power to address the hypothesis being tested?</br>
-Were correct statistical analysis used to support conclusions?</br>
-Are there concerns about ethical or regulatory requirements being met?</br></br>

Reviewer #1: No issues

Reviewer #2: The authors have improved the method section, however, I recommend to revise for minor details:

Line 122: add the units for the amount of cell-free bacteria that was inoculated.
We added the volume which we used to inoculated $5x10^7$ bacteria “in 200uL” on page 7 line 128.

Revise the subsection about "Suppression of innate immune activation by benidipine in vitro". I had to read it several times to understand it.
We provided more experimental details on the paragraph.

Line 213: What hBMECs stands for?
We added the complete name on page 12 line 235.

Reviewer #3: Yes

Results</br></br>
-Does the analysis presented match the analysis plan?</br>
-Are the results clearly and completely presented?</br>
-Are the figures (Tables, Images) of sufficient quality for clarity?</br></br>

Reviewer #1: The interpretation of the data that the levels of cytokines and chemokines are not significantly different with and without benidipine treatment does not seem to conform to what is shown in the figure, which shows increased gamma interferon and IL-6 with treatment. The *and ** are stated in the figure legend to indicate p<0.005 (is

p<0.05 what was really meant?) and p<0.001. These indeed would be significantly different.

Perhaps the figure and legend were not sufficiently clear, thus we have modified both. As stated previously in the legend and text, and despite the appearance of the box plots that represent median (line in middle of the box), quartiles 2 and 3 (25 and 75 percentiles boxes), minimum and maximum values (error bars), repeated statistical tests that used either Bonferroni, Tukey, or Holmes corrections demonstrate the lack of statistical significance when infected animals without benidipine treatment are compared to infected animals with benidipine treatment. The asterisks included on the image represented the significant differences in cytokine and chemokine concentrations when infected animals were compared to the matched (treatment or no treatment) mock infected animals. Because that designation was confusing, we have changed the image to directly show that comparisons of infected without treatment compared to infected with treatment are not significant, labeled by line and the designation "ns". These changes are also summarized in the figure legend.

"***Figure 10.*** *Plasma cytokine and chemokine concentrations after R. parkeri or mock-infection and treatment ± 3 or 10 mg/kg benidipine. Dramatic increases in concentrations are directly related to infection and are minimally modified by benidipine. Non-parametric t-tests with Bonferroni correction. While infection increased IFNγ, IL6, IL10, and all chemokines compared to mock infected animals treated or not with benidipine, statistically significant changes comparing infected animals without benidipine to infected animals which received either 3 or 10 mg/kg/d benidipine were not observed, noted here by "ns". Median values are the central line in the box that represents the 1st and 3rd quartiles; whiskers show 0 and 4th quartiles (min and max values).*"

Reviewer #2: The authors have improved the section, the new data and modification done to the figures allow a better understanding of the findings.

Reviewer #3: Yes for the texts, but I am still not happy with the excel quality figures. All the bar diagrams should be prepared in high quality without any gridlines, and show the statistical significance clearly.

It is a little difficult to discern to which figures the reviewer is referring. The graphs are generally constructed in either excel and customized as needed or in GraphPad, also customized. We have reviewed and optimized graphs and images on which reviewers commented and those that we feel could be further improved, in accordance with the limited reviewer suggestions. This includes revisions to Figures 1, 2, 4, 5, 6, 7, and 10, where excel graphs were improved for visibility and font size/appearance, or GraphPad graphs were improved to demonstrate the box plots and internal data points more clearly. Within the revised manuscript, jpg images are included to minimize file size, while all figures are submitted separately as tif images, as per instructions. Please note that the supplemental images are included as high resolution images that can be readily increased in magnification to examine cellular detail, including individual representative histopathologic lesions marked by arrows or arrowheads.

Conclusions</br></br>
-Are the conclusions supported by the data presented?</br>
-Are the limitations of analysis clearly described?</br>
-Do the authors discuss how these data can be helpful to advance our understanding of the topic under study?</br>
-Is public health relevance addressed?</br></br>

Reviewer #1: (No Response)

Reviewer #2: The authors have improved the section, the new data and analysis added valuable information that support the conclusions.

Reviewer #3: Yes

Editorial and Data Presentation Modifications?</br>
Use this section for editorial suggestions as well as relatively minor modifications of existing data that would enhance clarity. If the only modifications needed are minor and/or editorial, you may wish to recommend “Minor Revision” or “Accept”.

Reviewer #1: Revised manuscript would be improved for clarity by addressing the following:

1. Lines 44-46: It should be clarified that a retrospective review of patient's medications and outcomes are intended rather than a clinical trial.

While the existing language was not intended to encourage a clinical trial, we have clarified this to explicitly state the need for retrospective reviews of patient outcomes stratified by calcium channel blocker use.

2. Line 61-63: Pathogens should not be anthropomorphized. They have no intent.

We concur and have clarified this to hopefully reduce the potential for that interpretation.

3. Line 83: Does accelerated mean faster poor increased severity?

We have modified the sentence for additional clarity to indicate that the clinical signs occurred earlier and with greater severity, including early mortality.

4. Lines 125-127: Please fix the grammar. It is unclear where the first independent clause ends.

This sentence has been restructured for improved clarity.

5. Line 135: manufacturer's is the possessive case and requires an apostrophe.

Changed as suggested.

6. Lines 157 and 177: Please change the measure of concentration, micromolar, to the appropriate measure for thickness, micrometer.

Thank you for identifying this error. The changes have been made.

7. Line 193: The word performed would make sense rather than preformed.

This typographical error has been corrected.

8. Line 344: Is "reduction of white pulp" intended?

This has been changed to "loss of white pulp" for clarity.

9. Lines 353 and 502: What is the meaning of granuloma-like. I do not identify groups of activated macrophages.

The "granuloma-like" appearance is used to suggest the similarity of these small lesions to the initial stage of granuloma formation, where small numbers of histiocytes, both with an activated appearance, and others lacking that, accumulate. Without specific immunohistochemical identification, the specific cell type could be debated. However, we have carefully reviewed the lesions identified and maintain that the term is a good description of these lesions, and this is further meant to imply their response to differentiate lesions that do not mature to this appearance in the presence of benidipine. These lesions are clearly demonstrated in supplemental figure 3, where a single high magnification inset is shown, and where the lower magnification image demonstrates a range of these lesions that are located throughout the entire spleen of animals that did not receive benidipine.

10. Line 430: Is "cultured" meant?

Yes – thanks for identifying this typographical error.

11. Line 431: Is the correct word present or presence?

This word has also been corrected to "presence".

12. Line 153: Perivascular edema occurs in the brain in spotted fever group rickettsioses, but brainstem herniation requires a cited reference to confirm that it occurs.

While not routinely documented, cerebral herniation is a concerning complication of encephalitis with Rocky Mountain spotted fever, and is identified in many major texts.

To underscore this, we have added two case reports where herniation was documented.

13. Line 522: aut versus and

We have searched diligently to understand this comment and failed. No change made.

Reviewer #2: (No Response)

Reviewer #3: I am not sure if the production team can improve the figures, if yes, then they can do this.

As indicated above, we spent considerable time addressing improvements to the figures, especially the graphs. Please see comment above.

Summary and General Comments</br></br>
Use this section to provide overall comments, discuss strengths/weaknesses of the study, novelty, significance, general execution and scholarship. You may also include additional comments for the author, including concerns about dual publication, research ethics, or publication ethics. If requesting major revision, please articulate the new experiments that are needed.

Reviewer #1: The scientific rigor and contents of this manuscript are excellent.

Reviewer #2: The authors addressed the reviewers' main concerns and have improved the manuscript.

Reviewer #3: The authors have addressed most of my comments, and explained why they couldn't address some of the comments. Overall, the manuscript looks much better now. As mentioned above, I would suggest the authors to replace the Excel figures with high quality bar diagrams.

As indicated above, we spent considerable time addressing improvements to the figures, especially the graphs. However, as much of the data we have is NOT normally distributed, the use of bar graphs that show only mean and standard deviations is not appropriate. Thus, we used the appropriate graph style for such data, the box-plot that depicts the median as well as the 1st and 3rd quartiles, and minimum and maximum values. These graphs tend to be more busy, so we've tried to keep them as clean as possible.

PLOS authors have the option to publish the peer review history of their article (what does this mean?). If published, this will include your

full peer review and any attached files.

If you choose “no”, your identity will remain anonymous but your review may still be made public.

Do you want your identity to be public for this peer review? For information about this choice, including consent withdrawal, please see our Privacy Policy.

Reviewer #1: No

Reviewer #2: No

Reviewer #3: No
