# Supplementary material for: Benidipine impairs innate immunity converting sublethal to lethal infections in a murine model of spotted fever rickettsiosis
Source: PLoS Negl Trop Dis. 2024 Feb 26;18(2):e0011993. doi: 10.1371/journal.pntd.0011993 (PMC10919851; doi:10.1371/journal.pntd.0011993)
Supplement: Attachment — Submitted filename: 231227 reviews and comments.docx [file pntd.0011993.s012.docx]

| **Date:** | Oct 09 2023 07:28AM |
| --- | --- |
| **To:** | "J. Stephen Dumler" sdumler@jhmi.edu;john.dumler@usuhs.edu |
| **cc:** | "Andrés Felipe Londoño" pipelb@gmail.com, "Jennifer M. Farner" jennifer.farner.ctr@usuhs.edu, "Marlon Dillon" dillonm@mail.nih.gov, "Dennis J. Grab" dennis.grab@usuhs.edu, "Yuri Kim" yuri.kim.ctr@usuhs.edu, "Diana G. Scorpio" dscorpio71@gmail.com |
| **From:** | "PLOS Neglected Tropical Diseases" plosntds@plos.org |
| **Subject:** | Decision on your PLOS Neglected Tropical Diseases submission (PNTD-D-23-01130) |

Dear Dr. Dumler,
 
Thank you very much for submitting your manuscript "Benidipine stabilizes vascular permeability but impairs innate immunity in a murine model of spotted fever rickettsiosis" for consideration at PLOS Neglected Tropical Diseases. As with all papers reviewed by the journal, your manuscript was reviewed by members of the editorial board and by several independent reviewers. In light of the reviews (below this email), we would like to invite the resubmission of a significantly-revised version that takes into account the reviewers' comments.
 
We cannot make any decision about publication until we have seen the revised manuscript and your response to the reviewers' comments. Your revised manuscript is also likely to be sent to reviewers for further evaluation.
 
When you are ready to resubmit, please upload the following:

[1] A letter containing a detailed list of your responses to the review comments and a description of the changes you have made in the manuscript. Please note while forming your response, if your article is accepted, you may have the opportunity to make the peer review history publicly available. The record will include editor decision letters (with reviews) and your responses to reviewer comments. If eligible, we will contact you to opt in or out.

[2] Two versions of the revised manuscript: one with either highlights or tracked changes denoting where the text has been changed; the other a clean version (uploaded as the manuscript file).

Important additional instructions are given below your reviewer comments.

Please prepare and submit your revised manuscript within 60 days. If you anticipate any delay, please let us know the expected resubmission date by replying to this email. Please note that revised manuscripts received after the 60-day due date may require evaluation and peer review similar to newly submitted manuscripts.
 
Thank you again for your submission. We hope that our editorial process has been constructive so far, and we welcome your feedback at any time. Please don't hesitate to contact us if you have any questions or comments.
 
Sincerely,

Bin Gong, Ph.D., M.D.
Guest Editor
PLOS Neglected Tropical Diseases

Joseph Vinetz
Section Editor
PLOS Neglected Tropical Diseases

***********************


Reviewer's Responses to Questions

**Key Review Criteria Required for Acceptance?**


As you describe the new analyses required for acceptance, please consider the following:


**Methods**


-Are the objectives of the study clearly articulated with a clear testable hypothesis stated?

-Is the study design appropriate to address the stated objectives?

-Is the population clearly described and appropriate for the hypothesis being tested?

-Is the sample size sufficient to ensure adequate power to address the hypothesis being tested?

-Were correct statistical analysis used to support conclusions?

-Are there concerns about ethical or regulatory requirements being met?


Reviewer #1: Line 164: The molecular weight of the dextran to be stated here.
Line 180: The word " preformed" does not make sense.

Reviewer #2: Please see below

Reviewer #3: Yes, the methods used in this study are appropriate and statistical analyses were performed as necessary.

--------------------

**Results**


-Does the analysis presented match the analysis plan?

-Are the results clearly and completely presented?

-Are the figures (Tables, Images) of sufficient quality for clarity?


Reviewer #1: Lines 195 and 197: It does not make sense that animals were given a sublethal dose and became moribund

These animals were administered a dose estimate to be 1 LD_50_ within the 6 day interval of the model, as previously described by Londono et al (PLoS NTD) and in our pilot studies. That they were moribund, but had not died by day 6 was the endpoint we were seeking, and indicated that the estimate was not precise, but within reasonable range. That all mice treated 10 mg/kg/d and several treated with 3 mg/kg/d benidipine died earlier or had more severe infection showed the impact of the drug treatment. We have modified the text to more accurately reflect this design.
Line 223: It is more impressive that the organ titers were increased than the rickettsemia.

We agree that this is indeed impressive, but not necessarily surprising as the vast majority of bacteria are intracellular and bacteremia is a transient phenomenon, but is likely tissue burden-dependent.
Line 238 and throughout the results: The magnifications are insufficient to visualize the stated results. For example fibrinoid necrosis is not detectable at the magnification provided. The stated results are not questioned, but the statements that a large number of the lesions are visible in the figures are incorrect.

This is the reason we provided the high resolution images so that readers could examine both patterns of histopathologic injury at low magnifications and details of cellular injury at higher magnifications. If the reviewer did not see the supplemental high resolutions images, this would explain the comment. To include greater numbers of panels with each distinct pathologic finding we cite would be a significant use of space, but could be done if the reviewers and editors feel that detail is required.
Line 240: The statement is incorrect that meningitis is present in figure 3 C.

Agree. Panel C in Figure 3 was included as a “no pathology” control (rank 1) to contrast with the highest degree of brain histopathology in Panel D which was probably confusing. The text has been corrected.
Figure 3G: Are there any statistical differences in these values?

We re-examined the statistical evaluations of these values to assure that the proper approach for multiple comparisons of ranked data was used. In so doing, the application of post-hoc tests on significant Kruskal Wallis (non-parametric ANOVA) tests using the False Discovery Rate of Benjamani-Hochberg was applied to discern those significant pairwise comparisons. We include these p-values that are significant (<0.05) on the graphs in Figure 3. As can be seen, significant differences in pathologic severity existing when comparing infected (with or without benidipine) with mock infected (with or with benidipine) animals, generally at both days 3 and 6. In fact, severity did not differ among infected animals with or without benidipine in the lungs and brains, but our most important target organ, the liver, demonstrated the modulating impact of higher doses of benidipine on severity with R. parkeri infection.
Lines 255-259 and figure 5: Determination of vascular permeability in the liver is confounded by the discontinuous sinusoidal lining that includes both Kupffer cells and endothelial cells. Evaluation of vascular permeability in an organ with endothelial tight junctions such as the brain would be preferred. Agree – when brain and lungs were examined, there was no significant difference between benidipine treated and untreated infected mice. We modified the all aspects of the manuscript to reflect these new data and changes. We have replaced figure 5 with a summary graph showing the lack of effect of benidipine in vivo with *R. parkeri* sublethal infection.
Legend of figure 5. Hepatic loads were not determined here but rather fluorescence. The entirety of Figure 5 and the figure legend are changed, as described above.
Lines 260 and 262: "a modest potential therapeutic window" is stated redundantly.

Since the new analyses of brain and lung for vascular permeability were conducted, we have concluded that there is NO substantial effect of benidipine on vascular permeability in vivo, at least in the circumstance of *R. parkeri* infection.
Figure 6: No red arrows are visible.

All arrows have been enhanced for clearer visualization; this will also be available as a high resolution image for detailed inspection in the supplemental materials.

Line 313 and throughout the manuscript: The word fulminant indicates a rapid time of development. Its use to mean increased severity or mortality is incorrect.

Respectfully, the meaning also indicates an unusually severe course to the point of lethality. That the animals treated with benidipine died more rapidly and had significantly greater bacteremias despite similar inocula, we believe qualifies for the use of this term. However, since it appears that this definition is not shared by all SMEs, we have modified this to clarify our observations wherever the word “fulminant” was used previously.

Line 297: figures S3-S7 do not all demonstrate CD4 cells as the sentence states.

Agree and modified to reflect only supplemental figure 5 that shows CD4 cells.

Line 692: figure S8 was not provided in the manuscript.

We apologize for the error that was the result of mis-numbering. This has been corrected.

Reviewer #2: Please see below

Reviewer #3: Yes, the obtained results were presented well.

--------------------

**Conclusions**


-Are the conclusions supported by the data presented?

-Are the limitations of analysis clearly described?

-Do the authors discuss how these data can be helpful to advance our understanding of the topic under study?

-Is public health relevance addressed?


Reviewer #1: Line 355: It is unclear what is meant by "store-operated".

Store-operated calcium channels are a highly conserved group of calcium channels that are critical for entry of calcium into the cytoplasm of the cell from the external milieu for vital cell functions for both physiologic and pathophysiologic processes. Since this is a readily understood concept in channel biology, a further definition is not provided.

Reviewer #2: Please see below

Reviewer #3: I don't think the data presented fully support the claims including the title. Please see the general comments.

--------------------

**Editorial and Data Presentation Modifications?**


Use this section for editorial suggestions as well as relatively minor modifications of existing data that would enhance clarity. If the only modifications needed are minor and/or editorial, you may wish to recommend “Minor Revision” or “Accept”.

Reviewer #1: (No Response)

Reviewer #2: Please see below

Reviewer #3: NA

--------------------

**Summary and General Comments**


Use this section to provide overall comments, discuss strengths/weaknesses of the study, novelty, significance, general execution and scholarship. You may also include additional comments for the author, including concerns about dual publication, research ethics, or publication ethics. If requesting major revision, please articulate the new experiments that are needed.

Reviewer #1: This manuscript is an excellent contribution to the field of knowledge. In addition to many minor flaws, the discrepancy between what was observed and stated in the manuscript compared to what was visible in the figures is striking. This needs to be addressed effectively. The authors should recognize what features such as apoptosis and polymorphonuclear leukocytes are not visible in the figures.

This has been addressed by the addition of supplemental images and the use of arrows that help readers to identify the specific kinds of cells and pathologic patterns of injury that were observed through the review of multiple slides and immunohistochemical stains at a broad range of magnifications. It is not possible to completely provide the totality of the pathologic changes in images. Therefore, we have provided extensive representative documentation of the key findings in the figures with references to high resolution images that are provided as supplemental figures and described within the legends for these figures. We do hope that these images will be useful to readers.

Reviewer #2: The manuscript explores the repurposing of benidipine, a calcium channel blocker, to diminish the vascular dysfunction caused by Rickettsia parkeri in vivo in an experimental animal model of spotted fever rickettsiosis. Animals were treated with a sub-lethal dose of bacteria, followed by i.p. administration of different doses of benidipine.
Even though the manuscript described a decrease in vascular permeability, overall, the drug is not capable of reversing the effects of R. parkeri and in some parameters, the effects are enhanced after combining the drug with the bacteria. Likewise, the control group of uninfected mice treated with benidipine, displayed deleterious symptoms on the immune response (increased cellularity in splenic tissue of mice treated with 10 mg/kg/d of benidipine alone and release of inflammatory mediators) that debunk the primary hypothesis. The authors should not suggest the usage of this drug in infections by R. parkeri due to the adverse reactions it could cause.

This is a good interpretation of our analysis, but to be absolutely clear, our definitive interpretation is that this drug in such doses should NOT be advocated for use in this or similar conditions. Additionally, the further analysis of dextran leakage in the lung and brain provided no additional support for the use of benidipine for in vivo vascular permeability stabilization or reversal with vasculotropic rickettsioses such as in this model, and we have updated that section, our interpretations and the title to reflect this new information.

I consider the researchers needed to perform a pilot study of the drug itself, before conducting the actual experiments with the pathogenic bacteria, since the doses employed affected the groups in such a manner that the audience could question the whole experimental design. Regardless of the works cited by the authors to support the doses used, the strain of mice employed in this study is different as well as the drug’s administration route, compare to the studies repurposing the use of this drug for Chagas disease and estimating its acute toxicity, respectively, which could affect the final response seen in the controls.

We respectfully disagree that insufficient investigation was done prior to the study. The drug was safely used in preclinical studies in a range of circumstances, including infections, and found to be safe in the doses administered. We provided the references for these studies and also utilized the guidance of the IACUC in creating the protocols used, and all agreed with the plan. It is unfortunate that some doses had unanticipated adverse consequence even in the absence of infection, and especially so in the case of infected animals. However, it is just these kinds of studies that can reveal unique interactions that are not able to be studies in vitro.

In addition, the manuscript must be revised. It would be interesting to display the data of the mice uninfected but treated with 10/mg/kg/d of benidipine to see the alterations caused by the drug itself. Likewise, legends must be summarized and the discussion must be revised since the paragraphs are too long and key references are missing to support the ideas explained by the authors.

We are confused by the reviewer’s question since the data regarding mice uninfected and treated with 10 mg/kg/d is displayed throughout the manuscript, clearly identifying the lack of any substantial pathology. In accordance with journal specification and other reviewer requests, the legends include substantial detail such that the entirety of the figure can be explained within the legend. Also, the length of the paragraphs and total manuscript are within accordance with the journal’s guidance for authors, and without specific identification of “omissions”, revisions as requested are difficult. We have completely revised major components in accordance with reviewer’s comments, including new and more supportive references. We hope that these address the issues to which the reviewer has commented.

Reviewer #3: This article from Londono and colleagues report the unexpected impact of benidipine in impairing innate immunity while controlling the vascular permeability in a mouse model of spotted fever rickettsiosis. This is well written article and presents elegant data to demonstrate how benidipine exacerbates the infection by increasing bacterial load and altering the tissue morphology. However, the mechanistic details to demonstrate the effects of benidipine in affecting innate immunity specifically via calcium signaling is missing. Most of the results only present the actual observations in the animal model, not the actual mechanistic details to claim that benidipine is specifically affecting the innate immunity, possibly via intracellular calcium signaling. So, I would strongly suggest the authors to perform some additional experiments to demonstrate the direct effect of benidipine on immune cells in the presence and absence of R. parkeri. This could be done in culture cells if using animal models is tedious. If it is not possible to perform these additional experiments, then the authors should thoroughly edit this manuscript just to present the observational data and highlight the need for further studies in the discussion section to underpin the mechanistic details. They should also consider changing the title.

Thank you for the remarks and suggestions. We understood from the outset that the “descriptive” approach using pathology would not address mechanisms as well as we would have liked. Yet, the pathologic processes described here are known patterns established by long histopathologic and immunohistochemical analyses in murine and human tissues, including lymphoid tissues like spleen, that direct attention to the mechanisms as discussed. While the study was not powered, either by design or funding, to address the unanticipated immunologic dysfunction clearly shown by the pathology study, we have additionally taken the reviewer’s advice and studied immune signaling in innate immune lymphocytes (NKT cells) when stimulated by R. parkeri lysates in the presence or absence of benidipine. The utility of the innate immune NKT cells is that developing adaptive immune CD3, CD4 or CD8 T lymphocytes is not needed, and that NKT cells are CD3 cells and analyzed among those impacted by benidipine treatment in the animals studied here. The results of suppressed IL-2 production (the biomarker of NKT activation in the cell line we used) in the presence of benidipine is striking in its support for our hypothesis. This has been included in the manuscript and the discussion as further support, if not a solved mechanism that will be fodder for future investigations.

Some additional comments to consider while revising this manuscript.

1. There are some typographical errors in the introduction and discussion sections. Please correct them thoroughly.

We have carefully reviewed and corrected all other errors identified.

2. Some figures are presented in excel quality, which don't look great. So, I would strongly suggest the authors to prepare the final figures using another tool, and label all the axes clearly for all data.

We reviewed and prepared figures in accordance with this recommendation where apparent difficulties as described were detected. We hope that all have been addressed.

3. The figure legends are repeating the texts from results section. Instead, please only highlight the brief methods used to generate the data, and statistical analysis. There is no need to repeat the results in legends.

We streamlined the figure legends for those not presented as supplemental data and, in accordance with the instructions for authors, “avoiding lengthy descriptions of results, but providing “ the key messages” of the figure with a description “that will allow readers to understand it without referring to the text”. Additionally, we described “each part” of the “multipart figures with a lettered panel”, as per instructions.

4. In Figure 1B, please explain the missing line for 10 mg/kg in the legend. It would be helpful to show the statistical significance on this image somehow. For you could create a box/bar diagram for important data points.

We have revised the figure legend to indicate that all infected mice treated with 10 mg/kg/d benidipine died by day 3, and that only two of those treated with 3 mg/kg/d died by day 6. We have also modified the figure to more carefully identify significant changes and associated p values on panels B and C.

5. I am bit lost with the data presented in figure 3 because of the ranking system. I read several times, but couldn't completely understand the ranking system used. So please simply the text here and better explain this. Or the data can be presented similar to those in figure 4 and use the ranking system for the quantification but in a simple format.

We have simplified the rank system in the figure 3 legend, and it is explained in depth in the methods. This approach avoids the non-normal distribution that is derived from most semiquantitative measurements of histopathologic changes such that non-parametric statistical tests, that are more robust in these situations, can be applied.

6. Figure 5 - please create a better figure for quantified data, and include multiple comparisons between different panels.

Owing to extended analyses of dextran extravasation as a measure of vascular permeability using brain and lung in conjunction with liver, we determined that any value to benidipine protecting against increased vascular permeability in this in vivo model can not be supported, refuting one of the primary hypotheses. As a result, we created a new Figure 5 that demonstrates that quantitative measurement of dextran extravasation in all 3 tissues and each variable of the experimental infection only. All images that demonstrate the failure of benidipine to protect against increased vascular permeability in vivo with R. parkeri infection have now been moved into the supplemental data.

7. Figure 6 - panel B is really confusing. again, please use high quality figures and justify the staining for different markers.

The comment is confusing since panel A shows only H&E histopathologic changes in the spleen that depict the abnormal immune response reflected by the architecture of the splenic white matter and follicles, as well as the red pulp – both dynamic compartments with patterns of activation that correlate with effective or dysfunctional immune activation. However, there are no stains for different markers shown, only an enumeration of the relative quantities of specific and important innate and adaptive immune cells that characterize efficacy of the immune response in the spleen, the most important immune organ responding to blood stream infections. If the intent of the reviewer’s comment was to request justification as to why the specific cell immune phenotypes were selected, that has been added to the methods text. Please also note that the figures referred to for this section also include supplemental figures that are provided in high resolution to demonstrate specific features of importance. Because both architecture (low magnification) and cytology (high magnification) are required to identify the specific pathological changes, it is not readily possible to provide both in the main text figure. We selected what we believe demonstrates the main and obvious pathologic abnormalities and present the low magnification images to support the conclusions, as well as a quantitative approach to the specific changes in immune cell types in the spleen that determined immune and antimicrobial functions in supplemental figures.

8. Figure 8 and 9 - please see if you can provide some form of quantified data here.

While I understand the request for quantification of the IHC demonstration of R. parkeri, we do not feel this is needed for the following reasons: 1) PCR quantification, which is considerably more precise, was already conducted and presented earlier in the manuscript, including on liver, spleen, and brain tissues, and on whole blood; 2) quantification of R. parkeri in IHC is confounded because of the intracellular clustering in a manner that would not allow an accurate estimation of bacterial burden, since infected cells identified in this way could vary from a single bacterium in a cell to more than 100 bacteria per cell, two orders of magnitude for potential error. The real value of the IHC is to qualitatively and semiquantitatively demonstrate the tissue burdens in untreated vs. treated infected animals, and to discern to clustering of infected foci in the tissue. The PCR established definitive bacterial burden, while IHC shows the architecture of infection in specific tissues as it correlates with pathologic injury and immune/inflammatory cell infiltrates. To make this clearer, we have moved the R. parkeri PCR quantitation to the section in which R. parkeri IHC results are provided for their direct comparison.

9. Figure 10 - the data on cytokines and chemokines need better explanation. It's not clear how the statistical analyses were performed. I would suggest comparing all treated data with untreated controls and check the significance. Moreover please justify the use of these specific markers better. More mechanistic details are required for IL6, IL10 and RANTES as these were largely increased by benidipine.

Thank you for this comment as we agree that it lacks clarity. We have amended the legend for this figure to indicate that the ONLY statistically different cytokine/chemokine levels were comparing infected to uninfected animals at the same day p.i. and the same benidipine dose. The sole significantly different responses were limited to comparisons with uninfected mice treated with the same regimen of benidipine or no benidipine, and this has been clarified. Since cytokine and chemokine levels did not differ between infected or uninfected mice who received different benidipine doses, there are no p values presented. The specific cytokines and chemokines selected have been now justified in the methods section (lines 188-192).

10. Did the authors measure the blood parameters in these mice after treating with the drug? If yes, they will also demonstrate more mechanistic details about the impact of this drug.

This would have been optimal, but since the study did not anticipate such unprecedented consequences based on an extensive literature review and complete IACUC protocol review, it was not deemed necessary and therefore not powered to conduct that aspect of the study.

11. What sort of pathology was observed in dead mice due to high doses of benidipine? This will also add more to this study.

We agree that this too would have been highly revealing with regard to the 30 mg/kg/d dose, but unlikely revealing with regard to infection. However, as explained earlier, these animals died rapidly within 1-18h of receiving the drug, whether infected or not. Unfortunately, these animals were rapidly discarded and unable to be retrieved for examination. This was in part the result of very low staffing levels during the COVID pandemic when the studies were performed, and the misimpression that since it was clearly not infection related, that the process was not relevant.

--------------------

PLOS authors have the option to publish the peer review history of their article ([what does this mean?](https://journals.plos.org/plosntds/s/editorial-and-peer-review-process#loc-peer-review-history)). If published, this will include your full peer review and any attached files.


If you choose “no”, your identity will remain anonymous but your review may still be made public.


**Do you want your identity to be public for this peer review?** For information about this choice, including consent withdrawal, please see our [Privacy Policy](https://www.plos.org/privacy-policy).

Reviewer #1: No

Reviewer #2: No

Reviewer #3: No


Figure Files:
While revising your submission, please upload your figure files to the Preflight Analysis and Conversion Engine (PACE) digital diagnostic tool, https://pacev2.apexcovantage.com. PACE helps ensure that figures meet PLOS requirements. To use PACE, you must first register as a user. Then, login and navigate to the UPLOAD tab, where you will find detailed instructions on how to use the tool. If you encounter any issues or have any questions when using PACE, please email us at figures@plos.org.
 
Data Requirements:
Please note that, as a condition of publication, PLOS' data policy requires that you make available all data used to draw the conclusions outlined in your manuscript. Data must be deposited in an appropriate repository, included within the body of the manuscript, or uploaded as supporting information. This includes all numerical values that were used to generate graphs, histograms etc.. For an example see here: http://www.plosbiology.org/article/info%3Adoi%2F10.1371%2Fjournal.pbio.1001908#s5.
 
Reproducibility:
To enhance the reproducibility of your results, we recommend that you deposit your laboratory protocols in protocols.io, where a protocol can be assigned its own identifier (DOI) such that it can be cited independently in the future. Additionally, PLOS ONE offers an option to publish peer-reviewed clinical study protocols. Read more information on sharing protocols at https://plos.org/protocols?utm_medium=editorial-email&utm_source=authorletters&utm_campaign=protocols

__________________________________________________
In compliance with data protection regulations, you may request that we remove your personal registration details at any time. (Use the following URL: https://www.editorialmanager.com/pntd/login.asp?a=r). Please contact the publication office if you have any questions.
